# Influenza Vaccination Among Pregnant Women — Massachusetts, 2009–2010

**Published:** 2013-11-01

**Authors:** Renata Howland, Emily Lu, Hafsatou Diop

**Affiliations:** New York City Dept of Health and Mental Hygiene; Bur of Family Health and Nutrition, Massachusetts Dept of Public Health

The emergence of the novel influenza A (H1N1) pdm09 (pH1N1) strain in 2009 required a coordinated public health response, especially among high-risk populations. Because pregnant women were at increased risk for influenza-related complications and hospitalization compared with the general population (1), the American College of Obstetricians and Gynecologists and the Advisory Committee on Immunization Practices recommended pregnant women receive both the pH1N1 vaccine and the annual seasonal vaccine during the 2009–10 influenza season as a safe and effective way of protecting both mother and infant (2,3). To describe acceptance, predictors, and barriers to influenza vaccination among pregnant women in Massachusetts during the 2009–10 influenza season, the Massachusetts Department of Public Health (MDPH) analyzed data from supplemental influenza questions on the Massachusetts Pregnancy Risk Assessment Monitoring System (PRAMS) survey. The results indicated that 67.5% of residents who had live births in Massachusetts during September 2009–May 2010 received the seasonal vaccine, and 57.6% received the pH1N1 vaccine. Women who were non-Hispanic blacks, aged <25 years, Medicaid beneficiaries, or lived in a household with an income at or below the federal poverty level were significantly less likely to receive the seasonal vaccine. For the pH1N1 vaccine, only being non-Hispanic black was associated with being less likely to have been vaccinated. Vaccination rates were significantly higher among women whose provider offered or recommended the seasonal (75.8%) and pH1N1 (68.1%) vaccines compared with those who did not receive a recommendation (32.4% and 8.6%, respectively). Coverage in Massachusetts was among the highest of 29 PRAMS sites (4) and might have reflected strategic efforts by MDPH to support vaccine education and equity across the state (5).

Massachusetts PRAMS is a collaborative surveillance project between CDC and MDPH that collects state-specific, population-based data on maternal attitudes and experiences before, during, and shortly after pregnancy. Since 2007, the survey has been distributed to Massachusetts residents 2–6 months after delivery. Approximately 2,400 women are randomly selected to participate annually, with an oversampling of minority women to ensure adequate representation.* For the 2009–10 influenza season, MDPH added supplemental questions to the survey to gather information on state influenza vaccination coverage. A total of 1,038 women with live births during September 2009–May 2010 responded to the survey, with a weighted survey response rate of 65.1% in 2009 and 62.7% in 2010. Those with missing information on seasonal or pH1N1 vaccination (n = 42) were excluded from the analysis. The final sample included 996 women, representing 52,131 residents who gave birth in Massachusetts during September 2009–May 2010.

Women who indicated “yes” to the following questions were considered vaccinated: “Since September 2009, did you get a seasonal flu shot?” and “During your most recent pregnancy, did you get an H1N1 flu shot?” Various demographic and health service characteristics from PRAMS and the birth certificate were examined for their association with influenza vaccination acceptance, including age, race/ethnicity, education, Medicaid coverage, household income, nativity (born in the United States or elsewhere), primary language, and parity. Wald chi-squared tests were used to evaluate the statistical significance of select associations, and 95% confidence intervals were used to identify significant differences. Responses were weighted to represent all live births in Massachusetts, and all analyses were conducted using statistical software to account for the complex survey design and weighting.

During the 2009–10 influenza season, an estimated 67.5% of residents who had live births in Massachusetts received the seasonal influenza vaccine, and 57.6% received the pH1N1 vaccine (Table 1). Seasonal coverage was significantly lower among non-Hispanic black women (53.7%) compared with non-Hispanic white (69.6%) or non-Hispanic Asian (70.4%) women, and women who were aged <25 years (51.6%) compared with women who were aged 30–34 years (73.7%) or ≥35 years (79.2%). Seasonal coverage also was significantly lower among women who were Medicaid beneficiaries (57.3% versus 73.7%) or had a household income at or below the federal poverty level (56.1% versus 70.5%). For the pH1N1 vaccine, non-Hispanic black women were significantly less likely to report being vaccinated than were non-Hispanic Asian women (50.4% versus 65.5%). In contrast, women who received a provider recommendation were significantly more likely to receive the seasonal vaccine (75.8% versus 32.4%) and pH1N1 vaccine (68.1% versus 8.6%). The majority of women (71.7%) reported receiving the pH1N1 vaccine at their obstetrician-gynecologist’s office. Women also reported receiving the pH1N1 vaccine at their family doctor (11.7%), health department or clinic (8.0%), workplace, school, pharmacy (5.8%), or other locations (2.8%).

Among women who did not receive the seasonal vaccine, the most commonly cited reason was they did not normally get a flu shot (70.5%) (Table 2). Women also indicated they were worried about harm to their baby (43.0%) and side effects to themselves (37.5%). Women who did not get the pH1N1 vaccine reported greater worry about harm to their baby (52.8%) and side effects for themselves (50.6%). Another 46.0% of women reported that the pH1N1 influenza shot was unavailable, and 53.7% said they did not normally get a flu shot.

## Editorial Note

The findings in this report describe acceptance of vaccination by pregnant women in Massachusetts during the 2009–10 influenza season, along with predictors of and barriers to vaccination. Overall, Massachusetts had some of the highest rates of vaccination coverage among the 29 PRAMS states that collected this information. Compared with the median state coverage of 47.1% for seasonal and 40.4% for pH1N1, Massachusetts’s coverage was 67.5% and 57.6% respectively (4). Consistent with previous studies, there were significant racial/ethnic and socioeconomic differences between women who did or did not receive the seasonal vaccine, with lower rates among non-Hispanics blacks, Medicaid beneficiaries, and lower income women (6). However, fewer differences existed between women who did or did not receive the pH1N1 vaccine, possibly indicating some improvement in the methods used to promote the pH1N1 vaccine compared with the routine seasonal vaccine.

Provider recommendation was a significant predictor of acceptance, both for the seasonal and pH1N1 vaccine, contributing to high coverage statewide. In a study of pregnant women from Massachusetts General Hospital, 67% of women who received the pH1N1 influenza vaccine cited provider recommendation as the key factor that influenced their decision (7). In addition, the findings of this report indicate that safety concerns are a significant barrier to influenza vaccination, especially with the pH1N1 vaccine. These findings are similar to other studies, including a national poll in which concerns about the safety risks to baby and self were cited as the top reasons for not choosing to be vaccinated (8).

Specific actions from MDPH might have contributed to higher coverage among all pregnant women and fewer disparities in pH1N1 coverage. Soon after the outbreak began, officials dedicated more than $1 million to community-based organizations to work with racial/ethnic and linguistic populations who traditionally have lower rates of vaccination. They also provided resources for providers and clinics to support and encourage recommendations surrounding influenza vaccination. Lastly, MDPH developed a comprehensive Flu Facts media campaign that provided accurate, culturally appropriate information about influenza. These efforts became part of an ongoing focus on immunization equity across the state (5).

What is already known on this topic?

Vaccination rates improved during the influenza A (H1N1) pdm09 (pH1N1) outbreak in 2009, with variation across states and among population subgroups. Median coverage among 29 Pregnancy Risk Assessment Monitoring System (PRAMS) states was 47.1% for seasonal and 40.4% for pH1N1 influenza.

What is added by this report?

Data from the Massachusetts PRAMS survey indicated that during the 2009–10 influenza season, 67.5% of residents who had live births in Massachusetts received the seasonal vaccine and 57.6% received the pH1N1 vaccine. Non-Hispanic black women were less likely to receive either vaccine. Women who were aged <25 years, Medicaid beneficiaries, or from low-income households were significantly less likely to receive the seasonal vaccine. Vaccination rates were higher among women whose provider offered or recommended vaccination.

What are the implications for public health practice?

Targeted education and equity campaigns from the MDPH might have contributed comparatively high vaccination coverage rates and fewer disparities in pH1N1 coverage compared with seasonal vaccine coverage. Further efforts to promote the importance and availability of the influenza vaccine and to specifically address safety concerns could improve vaccination rates among pregnant women. Continued monitoring of vaccination coverage among pregnant women is needed to evaluate progress toward greater coverage.

The findings in this report are subject to at least four limitations. First, PRAMS is a self-reported survey administered 2–6 months after delivery; therefore, results might be subject to recall bias. Second, approximately 36% of women did not respond to the survey, and it is possible that weighting might not completely adjust for bias resulting from nonresponse. Third, the perceived availability of the seasonal vaccine was not included in the survey; therefore, no comparisons between seasonal and pH1N1 vaccine availability and the effect on coverage could be drawn. Finally, this analysis focused specifically on Massachusetts residents who had live births in Massachusetts and is not generalizable to pregnant women with different outcomes or in other states.

This report, using data from the PRAMS survey, presents a state-specific response to the emergence of a novel strain of influenza. Vaccination coverage in Massachusetts was high, with less variation among women who received the pH1N1 vaccine than among those who received the seasonal vaccine. Specific actions from MDPH to support vaccine education and equity across the state might have contributed to these patterns. These included supporting the role of providers, collaborating with community-based organizations, creating alternative sites to administer the vaccine, and developing culturally appropriate media campaigns. Efforts to promote the availability and importance of receiving the influenza vaccine and to specifically address safety concerns could further improve vaccination rates among pregnant women in Massachusetts. These findings can be used to encourage providers to recommend vaccination, address safety concerns, and engage community partners to increase vaccination acceptance in groups with low coverage. Continued monitoring of vaccination coverage among pregnant women is crucial to evaluate progress toward greater coverage.

## Figures and Tables

**TABLE 1:** Seasonal influenza and influenza A (H1N1) pdm09 (pH1N1) vaccination among pregnant women, by selected characteristics — Pregnancy Risk Assessment Monitoring System (PRAMS), Massachusetts, 2009–10 influenza season

	Seasonal influenza vaccination				pH1N1 influenza vaccination			
		
Characteristic*	No.	(%)†	(95% CI)	p-value	No.	(%)†	(95% CI)	p-value
**Total vaccinated**	**648**	**(67.5)**	**(63.4–71.3)**		**585**	**(57.6)**	**(53.4–61.8)**	
**Race**				<0.001				0.012
White, non-Hispanic	183	(69.6)	(63.7–75.2)		149	(57.3)	(51.1–63.2)	
Black, non-Hispanic	139	(53.7)	(47.5–59.8)		129	(50.4)	(44.1–56.6)	
Hispanic	161	(65.6)	(59.4–71.3)		147	(58.9)	(52.6–64.9)	
Asian, non-Hispanic	147	(70.4)	(63.7–76.3)		141	(65.5)	(58.8–71.7)	
**Age group (yrs)**				<0.001				0.044
<25	125	(51.6)	(42.8–60.3)		124	(50.3)	(41.5–59.0)	
25–29	182	(66.0)	(57.9–73.3)		158	(54.0)	(45.7–62.1)	
30–34	200	(73.7)	(66.7–79.7)		196	(65.8)	(58.2–72.6)	
≥35	141	(79.2)	(68.7–85.1)		107	(57.9)	(48.0–67.2)	
**Education (yrs)**				0.038				0.185
<12	65	(56.6)	(45.0–67.5)		71	(67.4)	(57.5–75.9)	
12	166	(63.1)	(54.4–71.1)		156	(55.7)	(47.0–64.1)	
>12	417	(71.1)	(66.0–75.5)		357	(57.3)	(51.9–62.4)	
**Medicaid**				<0.001				0.667
Yes	252	(57.3)	(50.6–63.7)		252	(56.7)	(50.1–63.1)	
No	390	(73.7)	(68.6–78.3)		331	(58.6)	(52.9–64.0)	
**Household income relative to federal poverty level** §				0.004				0.504
≤100%	146	(56.1)	(47.5–64.3)		143	(55.0)	(46.4–63.2)	
>100%	444	(70.5)	(65.6–75.0)		392	(58.4)	(53.1–63.4)	
**Nativity**				0.452				0.216
Non–U.S. born	324	(65.6)	(60.3–70.5)		310	(61.4)	(55.9–66.5)	
U.S.–born	323	(68.3)	(63.0–73.2)		275	(56.5)	(51.0–61.9)	
**Primary language**				0.274				0.700
English	547	(67.8)	(63.3–71.9)		488	(57.4)	(52.8–61.9)	
Spanish	71	(61.0)	(51.8–69.5)		72	(61.6)	(52.4–70.0)	
Other	30	(74.4)	(57.1–86.4)		41	(56.2)	(33.9–76.2)	
**Parity**				0.947				0.124
Primiparous	311	(67.3)	(61.4–72.7)		279	(54.3)	(48.1–60.3)	
Multiparous	335	(67.6)	(61.7–72.9)		304	(60.9)	(54.9–66.6)	
**Provider offered/Recommended**				<0.001				<0.001
Yes	589	(75.8)	(71.5–79.6)		573	(68.1)	(63.5–72.3)	
No¶	54	(32.4)	(23.9–42.3)		9	(8.6)	(4.1–17.4)	

**Abbreviation:** CI = confidence interval.

* The proportion of missing values were 3.0% (n = 30) for race, 0.2% (n = 2) for education, 0.2% (n = 2) for nativity, 1.0% (n = 10) for Medicaid, 8.7% (n = 87) for household income, 0.3% (n = 3) for parity, and 0.8% (n = 8) for provider offer/recommendation. Age, marital status, and primary language were not missing any observations.

† Weighted to adjust for complex survey design and nonresponse.

§ Household income relative to the federal poverty level was calculated using a combination of self-reported income and the number of dependent household members compared with 2009–2010 U.S. Department of Health and Human Services federal poverty guidelines. Because the exact dollar amount is not reported, the midpoint of each range was used to approximate household income.

¶ Small numbers (n <30) should be interpreted with caution.

**TABLE 2:** Barriers to vaccination among pregnant women who did not receive the seasonal (n = 348) vaccine or the influenza A (H1N1) pdm09 (pH1N1) vaccine (n = 411) — Pregnancy Risk Assessment Monitoring System (PRAMS), Massachusetts, 2009–10 influenza season

	Did not receive seasonal influenza vaccine			Did not receive pH1N1 vaccine		
		
Response	No.	(%)†	(95% CI)	No.	(%)†	(95% CI)
Doctor didn’t mention anything	80	(28.5)	(21.7–36.5)	72	(24.5)	(18.3–31.8)
Shot unavailable	—	—	—	114	(46.0)	(38.2–54.0)
Worried about side effects for me	106	(37.5)	(29.9–45.9)	166	(50.6)	(42.6–58.5)
Worried about harm to baby	118	(43.0)	(35.0–51.4)	157	(52.8)	(44.7–60.7)
I don’t normally get a flu shot	187	(70.5)	(63.0–77.0)	145	(53.7)	(45.7–61.6)
Other	63	(33.5)	(25.0–43.2)	51	(25.0)	(17.6–34.1)

**Abbreviation:** CI = confidence interval.

* Women could check more than one option; therefore, percentage will not total 100.

† Weighted to adjust for complex survey design and nonresponse.

## Footnotes

* Additional information on Massachusetts PRAMS is available at http://www.mass.gov/dph/prams.
